# Anticonvulsant activity of bone marrow cells in electroconvulsive seizures in mice

**DOI:** 10.1186/1471-2202-14-97

**Published:** 2013-09-06

**Authors:** Enéas Galdini Ferrazoli, Miriam Marcela Blanco, Simone Bittencourt, André Luis Lacerda Bachi, Luciana Bahia, Milena Botelho Pereira Soares, Ricardo Ribeiro-dos-Santos, Luiz Eugênio Mello, Beatriz Monteiro Longo

**Affiliations:** 1 Laboratório de Neurofisiologia, Departamento de Fisiologia, Federal University of São Paulo - UNIFESP, R. Botucatu, 862 5 andar, V. Clementino – CEP, 04023-066, São Paulo, Brazil; 2 Disciplina de Imunologia, Departamento de Micro-Imuno-Parasitologia, UNIFESP, São Paulo, Brazil; 3 Centro de Pesquisas Gonçalo Moniz, Fundação Oswaldo Cruz, Salvador, BA, Brazil; 4 Centro de Biotecnologia e Terapia Celular, Hospital São Rafael, Salvador, BA, Brazil

**Keywords:** Bone marrow, Cell transplantation, Electroconvulsive shock, Tonic seizure, Microglia, Hippocampus

## Abstract

**Background:**

Bone marrow is an accessible source of progenitor cells, which have been investigated as treatment for neurological diseases in a number of clinical trials. Here we evaluated the potential benefit of bone marrow cells in protecting against convulsive seizures induced by maximum electroconvulsive shock (MES), a widely used model for screening of anti-epileptic drugs. Behavioral and inflammatory responses were measured after MES induction in order to verify the effects promoted by transplantation of bone marrow cells. To assess the anticonvulsant effects of bone marrow cell transplantation, we measured the frequency and duration of tonic seizure, the mortality rate, the microglial expression and the blood levels of cytokine IL-1, IL-6, IL-10 and TNF-α after MES induction. We hypothesized that these behavioral and inflammatory responses to a strong stimulus such as a convulsive seizure could be modified by the transplantation of bone marrow cells.

**Results:**

Bone marrow transplanted cells altered the convulsive threshold and showed anticonvulsant effect by protecting from tonic seizures. Bone marrow cells modified the microglial expression in the analyzed brain areas, increased the IL-10 and attenuate IL-6 levels.

**Conclusions:**

Bone marrow cells exert protective effects by blocking the course of electroconvulsive seizures. Additionally, electroconvulsive seizures induced acute inflammatory responses by altering the pattern of microglia expression, as well as in IL-6 and IL-10 levels. Our findings also indicated that the anticonvulsant effects of these cells can be tested with the MES model following the same paradigm used for drug testing in pharmacological screening. Studies on the inflammatory reaction in response to acute seizures in the presence of transplanted bone marrow cells might open a wide range of discussions on the mechanisms relevant to the pathophysiology of epilepsies.

## Background

Epilepsy represents a complex group of disorders in which the main characteristic is the manifestation of spontaneous convulsive seizures that develop from specific areas in the central nervous system. From a clinical perspective, treatment to control seizures is restricted to anti-epileptic drug therapy. A growing number of reports have shown the therapeutic potential of stem cells obtained from various sources, such as bone marrow, brain, cord blood and skin, in the treatment of diseases affecting the central nervous system [1]. Bone marrow has been suggested as an accessible source of multipotent cells, and it has been used to treat patients with neurological diseases in a number of clinical trials [2-5]. It is believed that one of the major protective mechanisms of bone marrow stem cells is a paracrine effect by secreting substances with anti-inflammatory, anti-apoptotic, proliferative and pro-angiogenic actions [6-8]. However, bone marrow is also a source of inflammatory cells, which can infiltrate and proliferate into the brain parenchyma in response to alterations that may occur after epileptic seizures [9-13]. The continuous traffic of inflammatory cells into the cerebral tissue is accelerated after injury [14,15] and is involved in cytokine response to seizures [16-18]. Thus, the inflammatory reaction induced by acute seizure in the presence of transplanted adult bone marrow cells is a critical point that was evaluated here.

Maximum electroconvulsive shock (MES) is a classic experimental model for the induction of single generalized tonic-clonic seizure activity in rodents. MES mimics seizures commonly found in drug-resistant epilepsy patients, and it is the elected model for the primary screening of new antiepileptic drugs [19,20]. The MES screening test evaluates the effects of anticonvulsant drugs in preventing the spread of convulsive activity throughout the neural tissue, which is indicated by the abolishment of the hindlimb tonic extensor component of seizure [21,22]. Interestingly, the induction of MES has been implicated in alterations of microglial expression patterns and blood levels of cytokines, which indicates a commitment of inflammatory responses to the convulsive stimulus [23,24].

Based on these data, the focus of the current study was to investigate the effects of bone marrow cell transplantation on the behavioral and inflammatory responses to acute generalized tonic-clonic seizures induced by using the MES model. Because MES is the elected model to test anticonvulsive compounds, we proposed the use of MES to verify the ability of the transplanted bone marrow cells to inhibit seizure spread in the same paradigm as used in pharmacological screening tests.

## Results

### Behavioral seizure analysis

**Figure 1:**
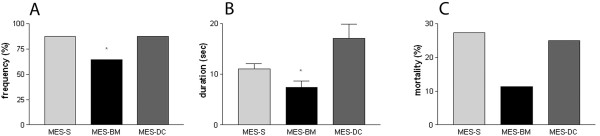
**Analysis of behavioral parameters of tonic seizures.** Percentage (frequency) of animals presenting with tonic seizures **(A)**; duration (seconds) of tonic seizures **(B)**; and the animal mortality rates (percentage) **(C)** between the three stimulated groups MES-S, MES-BM and MES-DC. The frequency of animals and duration of tonic seizures were significantly different and indicated reduction in theses parameters for the MES-BM group (*p < 0.05, Chi-Square test and ANOVA one-way followed by Tukey’s test). In **B**, data represent the means ± SEM.

MES stimulus (65 mA; 60 Hz; 0.15 sec) was applied in animals transplanted with bone marrow cells (MES-BM, n = 37), saline (MES-S, n = 35) and bone marrow dead cells (MES-DC, n = 12). Detailed analysis of the acute seizures induced by the MES stimulus indicated a reduction in the number of animals that developed tonic seizures with tonic hindlimb extension in the MES-BM group compared with the MES-S group (Chi-Square p = 0.039; ANOVA one-way followed by Tukey’s test, p = 0.0270). In the MES-S group, 88% of the animals and 83% in the MES-DC exhibited tonic seizures, whereas only 64% of the MES-BM transplanted animals had tonic seizures (Figure 1-A). Furthermore, the duration of tonic seizures was significantly reduced in MES-BM animals when compared to the MES-DC group (ANOVA one-way followed by Tukey’s test p = 0.0016; Kruskal-Wallis p = 0.0026; Figure 1-B). No differences were detected between MES-S and MES-DC. We also observed a lower mortality rate among animals in the MES-BM group (12%) compared with the MES-S and MES-DC groups that doubled the value (25%), although this difference was not statistically significant (Figure 1-C).

### Microglial analysis

The microglia expressing Iba1 was analyzed in naïve medium- and BM-injected animals (CTRL), MES-S, MES-BM groups in the neocortex (Cx), striatum (CPu), hippocampus (Hp), substantia nigra (SN), superior colliculus (SC), and periaqueductal gray area (PAG). Naïve bone marrow-injected animals composed, together with medium-injected animals, the control group for the Iba1 quantification (CTRL). Differences between the groups were detected for three main regions of interest, Cx, Hp and SN based on evidence that these areas are involved in motor activity [25] and electroconvulsive seizure susceptibility [26-28].

**Figure 2:**
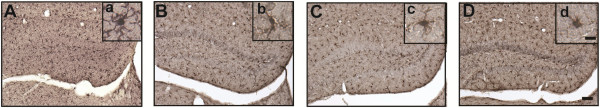
**Photomicrographs showing representative sections of Iba1-stained cells in the hippocampus of the four groups, medium-injected naïve CTRL (A), MES stimulates saline-injected (MES-S) (B), MES stimulated bone marrow-injected (MES-BM) (C), and MES stimulated dead cell-injected (MES-DC) (D) animals.** Note the different morphological changes of Iba1 cells in a control animal (a) that show intense arborization of microglial cells compared with animals exposed to MES (S, BM and DC, respectively in b, c and d). Scale bars represent 100 μm **(A-D)** and 10 μm (a-d).

Qualitative analysis of Iba1 cells indicated that there were morphological differences between cells in the non-stimulated control (CTRL = medium and BM) and the MES groups. In CTRL group, Iba1 cells displayed typical resting microglia morphology, were densely ramified with thin processes [29,30]. The Iba1 cells of MES-S, MES-DC and MES-BM stimulated animals displayed thicker and fewer ramifications of cell processes compared to non-stimulated animals. This morphology has been described as an intermediate stage between resting and reactive microglia [31,32] (Figure 2, A-D, inset a-d).

**Figure 3:**
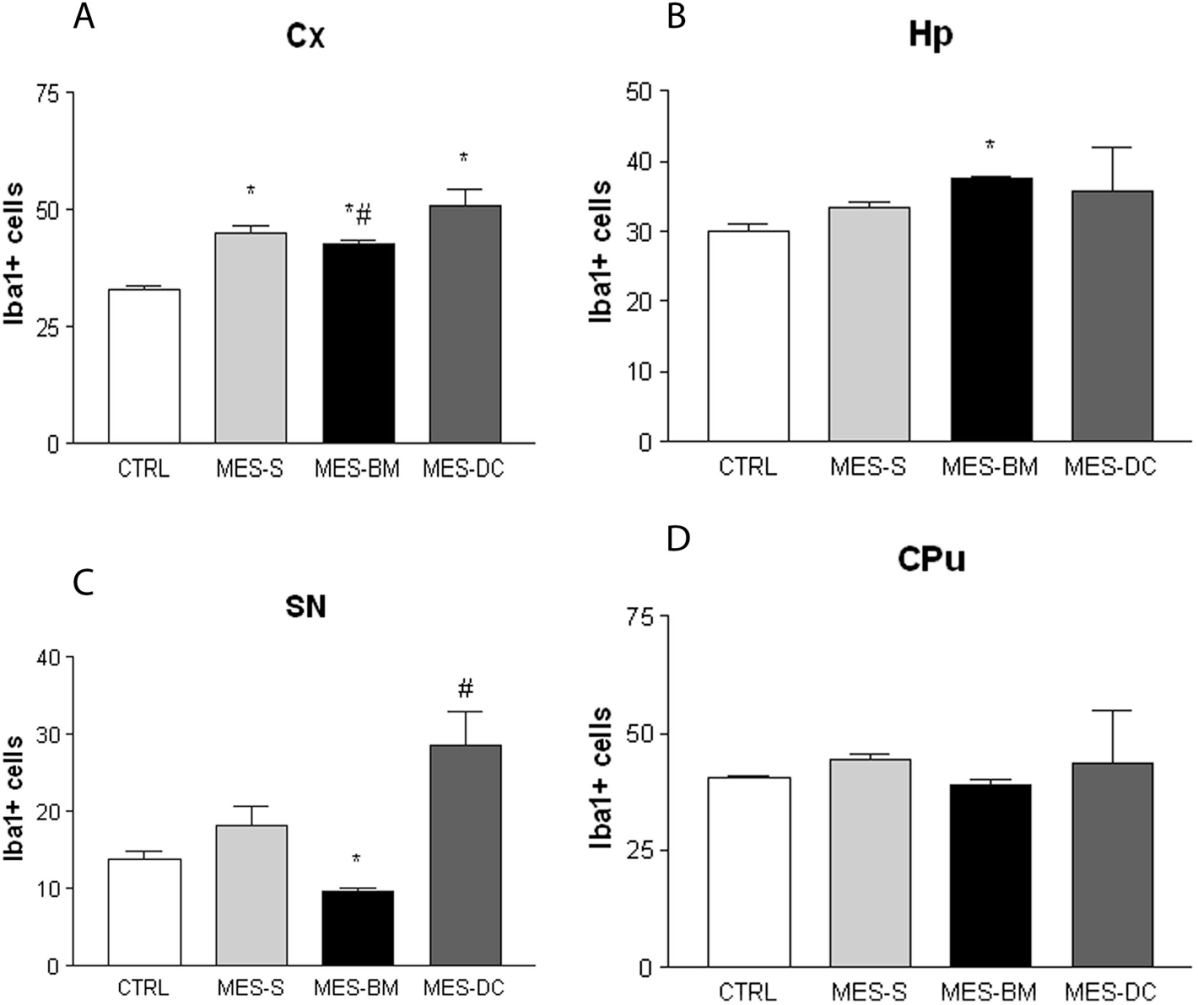
**Quantification of microglia expressing Iba1 in four brain structures of naïve medium- and bone marrow-injected CTRL, MES-S, MES-BM and MES-DC animals: (A) cortex (Cx); (B) hippocampus (Hp); (C) substantia nigra (SN); and (D) striatum (CPu).** A significant reduction in the number of Iba1 cells was detected in the Cx **(A)**, Hp **(B)** and SN **(C)** for f the MES-BM group (In **A** *p < 0.05 versus CTRL group; #p < 0.05 versus MES-DC group; in **B** *p < 0.05 *versus* CTRL and MES-DC groups; in **C** *p < 0.05 *versus* the three groups; #p < 0.05 *versus* CTRL). ANOVA one-way followed by Tukey’s multiple comparison test and Kruskal-Wallis, followed by Dunn’s test. Data are presented as the mean ± SEM.

The quantitative analyses of Iba1 cells in the Cx of the animals in the MES groups showed a higher values of Iba1 cells compared with CTRL (ANOVA one-way followed by Tukey’s test, p < 0.0001). MES-BM group showed a lower number of cells expressing Iba1 when compared to MES-DC animals (Tukey’s test, p < 0.05; Figure 3-A). In the Hp, Kruskal-Wallis followed by Dunn’s test detected differences between MES-BM and CTRL, and MES-BM and MES-DC (p = 0.0004), indicating that the number of Iba1 cells in MES-BM-treated group was higher when compared to these groups (p < 0.05; Figure 3-B). Differences were also found for SN indicating a significant reduction in the number of Iba1 cells of the animals in MES-BM compared to MES-S, MES-DC and CTRL groups (ANOVA one-way followed by Tukey’s test, p < 0.0001; Figure 3-C). Although MES-BM showed lower values for quantification of positively-stained Iba1 cells in the CPu, SC and PAG, no significant difference was detected between the groups in these areas (data are not shown for the SC and PAG).

### Cytokine analysis

**Figure 4:**
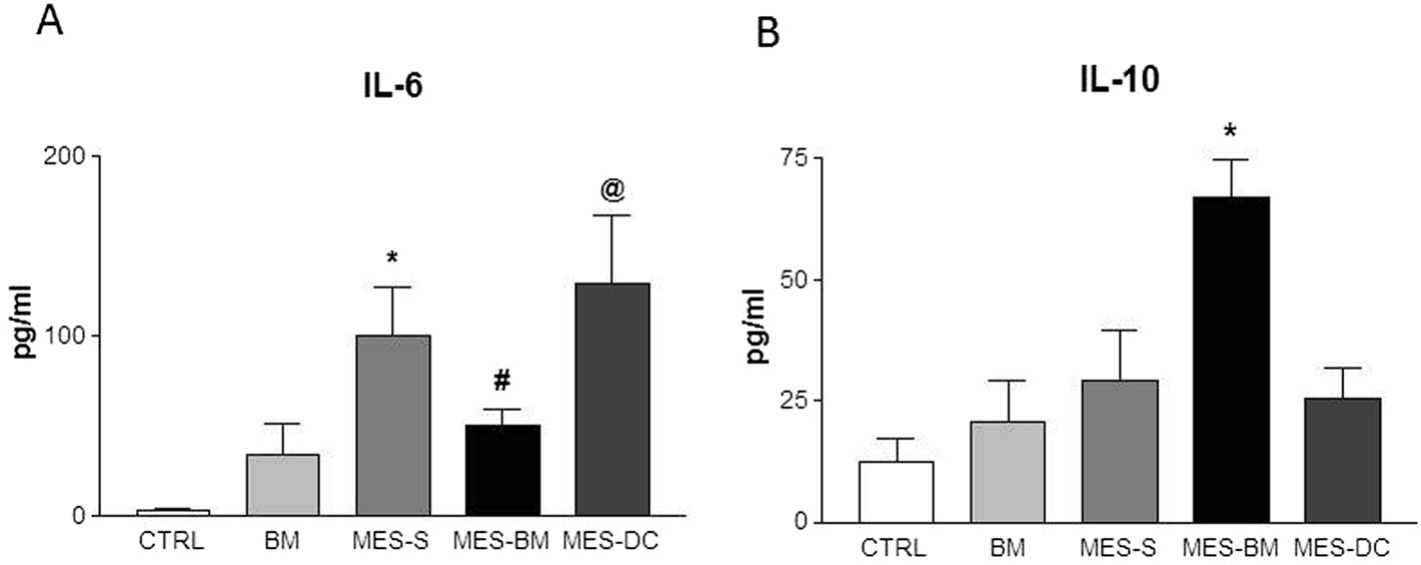
**Histograms depicting the blood levels of IL-6 (A) and IL-10 (B) of naïve CTRL medium- and BM-injected animals and, MES-S, MES-BM and MES-DC animals.** ANOVA one-way followed by Tukey’s multiple comparison test indicates significant differences (in **A** *p < 0.05 *versus* CTRL and BM; ^#^p < 0.05 *versus* MES-DC; ^@^p < 0.05 *versus* CTRL, BM and MES-BM groups; in **B** *p < 0.01 *versus* the four groups). Data represent means ± SEM.

To investigate the mechanisms by which bone marrow cell therapy resulted in a protection of tonic seizures, we tested whether bone marrow cell transplantation modulates the levels of IL-1, IL-6, IL-10 and TNF-α. Blood samples drawn to quantify levels of cytokines indicated that IL-6 levels were diminished in MES-BM group compared to MES-DC (ANOVA one-way p = 0.0006, followed by Tukey’s test, p < 0.05). Also, significant difference was detected when both experimental groups S and BM was compared (Student’s t test p = 0.0298). Although higher IL-6 levels were detected in both the MES-S and MES-DC groups when compared to naïve medium and BM-injected CTRL groups (p < 0.05; p < 0.01; Figure 4-A), no difference was found between MES-BM and CTRL or BM groups.

In contrast, for IL-10 blood levels, we detected a higher value for the MES-BM group compared to MES-S, MES-DC and naïve CTRL and BM groups (ANOVA one-way, followed by Tukey’s test p < 0.0001; t test MES-S *versus* MES-BM p = 0.0148; Figure 4-B). The levels of IL-1α, IL-1β and TNF-α did not differ significantly between the MES groups submitted to the different treatments (S, DC or BM) or CTRL (data are not shown for the IL-1α, IL-1β and TNF-α).

Correlation analyses were performed to assess the relation of the duration of tonic seizure with the number of Iba1 cells and the blood levels of IL-6 and IL-10. Significant positive correlations were noted between the tonic duration and the number of Iba1 in the cortex (r = 0.9998; p < 0.0001), and between tonic duration and IL-6 (r = 0.9167; p = 0.0165). The longer the tonic seizure the more Iba1 cells counted in the cortex, and the higher the IL-6 blood levels. The same was near to occur between the tonic duration and IL-10, with the difference coming close to the significance threshold for a negative correlation (r = − 0.7594; p = 0.0799). No other significant correlation was observed.

## Discussion and conclusions

Our results indicate that bone marrow cells may alter the convulsive threshold and also show anticonvulsant effects by protecting transplanted animals from tonic seizures. Bone marrow cells were tested under the same paradigm as that employed in the pharmacologic screening of potential antiepileptic drugs [21], using MES as a model to evaluate the protective effect of the cells *in vivo*. As suggested by Stable and Kupferberg [33], the ability of the tested compound to inhibit MES-induced seizure spread is indicated by abolition of the hindlimb tonic extensor component of seizures. With this experimental design, primary screening showed that bone marrow cells were able not only to protect transplanted animals against the tonic component, but also to reduce the duration of the tonic phase of generalized seizures. These data are consistent with previous findings in which authors observed a protective effect of bone marrow cells transplanted in already-epileptic animals [34].

As has been outlined before, bone marrow-derived cells in the CNS contribute mainly for the generation of microglia [14,35-40]. We reported previously that after *status epilepticus*, the number of microglia originated from bone marrow increases dramatically [12]. However, here the temporal window between cell transplantation and the electroshock stimulus was too short to have allowed for migration of the cells into the brain parenchyma. Indeed, after the electroshock GFP^+^ cells were not present in the parenchyma of animals transplanted with bone marrow cells obtained from the transgenic EGFP donor mice (data not shown). We speculate that bone marrow transplanted cells might have a paracrine action that influences the microglial activity, in areas such as cortex, substantia nigra and hippocampus, and the release of modulatory substances that could switch off the tonic activity. As already described, microglia have been observed to show morphological alterations after electrical convulsive shock [23,24]. Jansson and colleagues described a reduction of the density of processes of microglia following electroconvulsive shock. In a similar manner, we observed a clear reduction in the ramification of resident microglia after the electrical convulsive stimulation, regardless of the bone marrow cell administration, indicative of an intermediate stage between resting and reactive microglia [31,32].

As suggested by Browning and colleagues, generalized seizures have distinct neuroanatomical substrates that start and sustain the seizure activity [41]. After the electroconvulsive shock, the reactive response of microglia in the striatum and substantia nigra, areas strongly connected and related to motor activity, is probably due to the intense motor activity seen during the tonic seizure. Microglial expression was otherwise suppressed in transplanted animals (MES-BM compared with MES-DC) in SN (with a tendency in CPu) because the motor activity related to tonic seizure was protected by bone marrow cell transplantation. Although the tonic component of seizure was protected in MES-BM animals, the stimulated groups (MES-S, MES-DC and MES-BM) presented clonic seizures, which may explain the absence of differences in these areas and consequently suggest their responsiveness to the clonic seizure. In the hippocampus, a structure that is susceptible to convulsive seizures [42,43], and that contains cells vulnerable to electroshock [26-28], transplantation of bone marrow cells in MES animals was able to raise the number of microglial cells. Concerning these data, we speculated whether the observed increase in Iba1 cells in the hippocampus was due to its role in generalized or focal seizures and/or by a direct participation on persisting clonic seizures. As proposed by Silverberg and co-authors, inflammatory cells are specifically recruited to damaged regions of brain associated with the focus of seizure [44]. These authors suggested that in MES seizures, currents may spread throughout the brain and the intensity and/or duration of stimulus could alter cytokine and chemokine production. It has been also described that the number of Iba1cells was increased around the mesenchymal bone marrow transplanted cells in hippocampus of mice with Alzheimer’s disease [45]. The transplantation *per se* accelerates the activation of microglia, regardless of pathological condition. In our case, the generalized seizures promoted by the MES stimulation, together with the injection of BM cells, may suggest that the higher numbers of hippocampal microglia found in transplanted animals function as part of an inflammatory response to seizure.

As suggested by Jankowsky and Patterson [32], the onset and duration of seizure are influenced by a variety of types and levels of cytokines that, at the time of induction, may compete to determine the behavioral outcome. Several studies have reported elevated concentrations of IL-6 immediately after seizure activity [46,47]. In our hands, the trigger of MES convulsion induced an increase in IL-6 blood levels in both MES-S and MES-DC groups. However, these levels were decreased in animals transplanted with bone marrow cells. The low levels of IL-6 detected in MES-BM mice might be related the reduction of tonic seizure in these animals, as suggested by Pearson’s correlation. In concert with the decrease in IL-6 levels observed in BM transplanted animals, blood levels of IL-10 were higher in those animals, which may reduce susceptibility to seizures induced by pro-inflammatory cytokines, although it was not detected in correlation analysis. Our data corroborate other findings that showed a reduction in the pro-inflammatory cytokines and an increased in the IL-10 of chronic epileptic rats treated with mouse bone marrow cells [48]. Accordingly, other authors proposed that IL-10 expression may be induced simultaneously with pro-inflammatory cytokines in the brain following an insult [49,50]. The beneficial or detrimental roles of the innate immune response in the epileptic tissue, however, still needs to be clarified [17]. To understand the relationship between cytokine signaling and bone marrow cell function in the epileptic brain, additional studies during the chronic period of spontaneous seizures are required.

Taken together, these results suggest that transplanted bone marrow cells exert protective effects by blocking the tonic component of generalized seizures induced by MES. Moreover, electroconvulsive seizures applied in transplanted mice triggered acute responses altering the pattern of microglia expression and blood levels of interleukins. Additionally, we suggest that the anticonvulsive potential of progenitor cells may be tested by the MES model, as a primary screen, in the same paradigm as that used for drug testing in pharmacological screening. In spite of these promising results, several considerations concerning the transplantation strategies, such as the number of cells and the timing and route of administration should be elucidated. Further studies on the inflammatory response to acute seizures in the presence of transplanted bone marrow cells could prove to be beneficial, and may open broad and productive discussions on mechanisms relevant to the pathophysiology of epilepsy.

## Methods

All animals were housed in a pathogen-free facility and maintained in accordance with the Guide for the Care and Use of Laboratory Animals (National Research Council). All protocols were approved by the Ethics Committee of Universidade Federal de São Paulo (UNIFESP), process 0507/07, conformed to current guidelines and with approval by the Comissão Nacional de Ética em Pesquisa (CONEP).

### Bone marrow cell extraction

Bone marrow cells were obtained from adult C57Bl/6-donor mice (20–25 g) (JAX® Mice and Services, Bar Harbor, Maine, USA) by flushing the femurs and tibiae with sterile medium. The mononuclear cell fraction was purified by centrifugation in a Ficoll-Paque gradient (Stemcell Technologies Inc, Vancouver, Canada). The cells were washed four times in Dulbecco’s Modified Eagle Medium (DMEM, Gibco, Grand Island, NY, EUA), counted and resuspended in sterile saline. Viability of cells was determined by trypan blue (Gibco, Grand Island, NY, EUA) dye exclusion test. Briefly, cells were incubated with trypan blue dye for 1 min. White cells was counted excluding blue positive cells in Neubauer chamber, and the percent of viable cells was calculated. Approximately 1 × 10^6^ cells diluted in 200 μL or saline were delivered to each recipient animal by intravenous administration. Additionally to the saline (MES-S) and bone marrow (MES-BM) groups, age-matched groups were prepared by injecting similar volumes of bone marrow dead cells (MES-DC), obtained by repeated frost and thaw cycles, as a control for the inflammatory response, conditioned medium and cell toxicity; naïve bone marrow alive cells (BM), and medium-injected (CTRL) groups that were not seizure-induced by MES.

### Maximum electroconvulsive shock (MES) induction

Thirty minutes after saline or cell administration, an electrical stimulus (65 mA; 60 Hz; 0.15 sec duration) was applied through a pair of corneal electrodes (AVS, Solução Integrada, São Paulo-SP) to induce acute generalized tonic-clonic seizure [19,20]. The criterion for the occurrence of seizure activity was the tonic hindlimb extension response. The parameters used to estimate the anticonvulsant activity of transplanted cells were the protection against tonic hindlimb extension and the reduction in tonic seizure duration.

### Immunohistochemistry

Two hours after the MES stimulus, mice were intraperitoneally anesthetized with thionembutal (200 mg/kg; Cristália, Brazil) and transcardially perfused with phosphate buffered saline (PBS; 100 mM, pH 7.2), followed by 4% paraformaldehyde (PFA) in PBS (w/v), 50–100 mL over 10 minutes per animal. The brains were removed and processed for immunohistochemistry on free-floating brain slices. Coronal brain sections (30 μm thick) were made between bregma 0.98 and bregma −3.28 mm, targeting the areas of interest in forebrain and midbrain; Cx, CPu, Hp, SC, SN, and PAG, according to the stereotaxic coordinates of the mouse brain atlas [51]. The sections were selected (9 per animal) by targeting the areas of interest (neocortex, striatum and hippocampus), which were left free-floating in a multi-well plate. To stain microglia, sections were incubated in polyclonal rabbit anti-Iba1 primary antibody overnight (1:2000; Wako Chemicals, Richmond, VA, USA), followed by a biotinylated anti-rabbit secondary antibody for 2 h (1:600, Sigma-Aldrich Corporation, St. Louis, EUA). After using an ABC kit (Avidin/Biotinylated enzyme Complex Vectastain Elite, Vector, Burlingame, CA, EUA) followed by a DAB reaction (3,3’-Diaminobenzidine, Sigma-Aldrich Corporation, St. Louis, EUA), the sections were mounted on slides and sealed with coverslips. The slides were examined using a light microscope (Nikon 80i), and images were captured and digitized using the Nikon ACT-1 v.2 system. In each section, nuclear profiles of Iba1 cells were counted by an observer blinded to the experimental condition of each animal. Cells stained for Iba1 were counted under 20× magnification in 18 random, non-overlapping fields of 0.02 mm^2^ per structure of each animal.

### Cytokine analysis

With the animals deeply anesthetized, approximately 1 mL of blood was collected from abdominal aorta artery just before cardiac perfusion. Blood samples placed in 1.5 mL tubes were allowed to clot for 2 h at room temperature before centrifugation for 15 min at 1000 × g. The sera were collected, aliquoted and stored at −20°C. Analyte-specific antibodies against IL-1α, IL-1β, IL-6, IL-10 and TNF-α were pre-coated onto color-coded microparticles using the MAP Fluorokine kit (R & D Systems, Inc.). Microparticles, standards and samples were pipetted into wells and incubated with antibodies specific for the analytes of interest. After washing, streptavidin-phycoerythrin conjugate (Streptavidin-PE) was added to each well. The microparticles were resuspended in buffer and quantified using the Luminex analyzer (Luminex® 100/200 System, Austin, USA).

### Statistics

Statistical analyses were performed using Prism software (version 288 5.01, GraphPad Software, San Diego, CA, USA). Behavioral seizure, Iba1 cell and cytokine quantifications were analyzed with one-way analysis of variance (ANOVA) followed by Tukey’s multiple comparison test, and confirmed with Kruskall-Wallis followed by Dunn’s multiple comparison test. Student’s t test was used to compare two experimental groups. A significance level of 5% was assumed.

## Competing interests

The authors declare that they have no competing interests.

## Authors’ contributions

EGF conceived the study, carried out the laboratory experiments, analyzed the data and performed the statistical analysis; MMB participated in the experimental design and helped to collect the data and draft the manuscript; SB carried out the immunohistochemistry and cell quantification and prepared the figures; ALB carried out the interleukin analysis and critically revised the paper; LMB carried out the laboratory experiments, data collection and immunohistochemistry; MBS helped to draft the manuscript, to interpret the results and critically revised the paper; RRS and LEM helped with the general idea of the paper; contributed with the reagents/materials/analysis tools, and critically revised the paper; BML defined the research theme, conceived and designed the experiments and the general idea of the paper, interpreted the results and wrote the paper. The work presented here was carried out in collaboration between all authors. All authors read and approved the final manuscript.
